# Supplementary material for: Mapping the evolution of fertility support policies in China: A content and instrumental analysis
Source: PLoS One. 2025 Oct 9;20(10):e0332137. doi: 10.1371/journal.pone.0332137 (PMC12510515; doi:10.1371/journal.pone.0332137)
Supplement: S1 Appendix — (ZIP) [file pone.0332137.s001.zip › S1 Appendix. 226 original policy documents/185-国家发展改革委关于印发《2020年新型城镇化建设和城乡融合发展重点任务》的通知(FBM-CLI-4-341396).doc]

国家发展改革委关于印发《2020年新型城镇化建设和城乡融合发展重点任务》的通知

**发布部门：** [国家发展和改革委员会(含原国家发展计划委员会、原国家计划委员会)](https://www.pkulaw.com/law/chl?Aggs.IssueDepartment=60301) [机构沿革](javascript:void(0))

**发文字号：**发改规划〔2020〕532号

**发布日期：**2020.04.03

**实施日期：**2020.04.03

**时效性：** [现行有效](https://www.pkulaw.com/law/chl?Aggs.TimelinessDic=01)

**效力级别：** [部门规范性文件](https://www.pkulaw.com/law/chl?Aggs.EffectivenessDic=XE0302)

**法规类别：** [城市规划与开发建设](https://www.pkulaw.com/law/chl?Aggs.Category=04903)

国家发展改革委关于印发《2020年新型城镇化建设和城乡融合发展重点任务》的通知
（发改规划〔2020〕532号）

中央和国家机关有关部门、直属机构，各省、自治区、直辖市及计划单列市、新疆生产建设兵团发展改革委，全国工商联，中国国家铁路集团有限公司、国家开发银行、中国农业发展银行：
　　经城镇化工作暨城乡融合发展工作部际联席会议成员单位共同确定并报告国务院，现将《2020年新型城镇化建设和城乡融合发展重点任务》印发你们，请认真贯彻执行。

国家发展改革委
2020年4月3日

　　2020年新型城镇化建设和城乡融合发展重点任务

　　2020年是全面建成小康社会和“十三五”规划收官之年，也是为“十四五”发展打好基础的关键之年。为深入贯彻落实习近平总书记关于统筹推进新冠肺炎疫情防控和经济社会发展工作的重要指示精神，贯彻落实中央经济工作会议精神和党中央、国务院印发的《国家新型城镇化规划（2014-2020年）》《[关于建立健全城乡融合发展体制机制和政策体系的意见](https://www.pkulaw.com/chl/d3cf3bbf007959eebdfb.html)》，现提出以下任务。
　　一、总体要求
　　以习近平新时代中国特色社会主义思想为指导，全面贯彻党的十九大和十九届二中、三中、四中全会精神，坚持稳中求进工作总基调，坚持新发展理念，加快实施以促进人的城镇化为核心、提高质量为导向的新型城镇化战略，提高农业转移人口市民化质量，增强中心城市和城市群综合承载、资源优化配置能力，推进以县城为重要载体的新型城镇化建设，促进大中小城市和小城镇协调发展，提升城市治理水平，推进城乡融合发展，实现1亿非户籍人口在城市落户目标和国家新型城镇化规划圆满收官，为全面建成小康社会提供有力支撑。

　　二、提高农业转移人口市民化质量
　　以深化改革户籍制度和基本公共服务提供机制为路径，打破阻碍劳动力自由流动的不合理壁垒，促进人力资源优化配置。
　　（一）督促城区常住人口300万以下城市全面取消落户限制。督促Ⅱ型大城市和中小城市（含设区市和县级市）坚决贯彻《中共中央办公厅 国务院办公厅关于促进劳动力和人才社会性流动体制机制改革的意见》，全面取消落户限制，进一步促进劳动力和人才社会性流动。（公安部、发展改革委、省级有关部门等负责）
　　（二）推动城区常住人口300万以上城市基本取消重点人群落户限制。督促除个别超大城市外的其他超大特大城市和Ⅰ型大城市坚持存量优先原则，取消进城就业生活5年以上和举家迁徙的农业转移人口、在城镇稳定就业生活的新生代农民工、农村学生升学和参军进城的人口等重点人群落户限制。推动Ⅰ型大城市探索进城常住的建档立卡农村贫困人口应落尽落。鼓励有条件的Ⅰ型大城市全面取消落户限制、超大特大城市取消郊区新区落户限制。（公安部、发展改革委、省级有关部门等负责）
　　（三）促进农业转移人口等非户籍人口在城市便捷落户。鼓励各城市政府简化户籍迁移手续，加强落户政策宣传，开通线上申请审核系统，大幅提高落户便利性。推动超大特大城市和Ⅰ型大城市改进积分落户政策，确保社保缴纳年限和居住年限分数占主要比例。（公安部、发展改革委、省级有关部门等负责）
　　（四）推动城镇基本公共服务覆盖未落户常住人口。出台国家基本公共服务标准。提高居住证发证量和含金量，推动未落户常住人口逐步享有与户籍人口同等的城镇基本公共服务。运用信息化手段建设便捷高效的公共服务平台，加快养老保险全国统筹进度，完善基本医疗保险跨省异地就医医疗费用直接结算制度，做好社会保险关系转移接续，方便人口流动。增加学位供给，健全以居住证为主要依据的随迁子女入学入园政策，使其在流入地享有普惠性学前教育。以解决新市民住房问题为主要出发点，完善住房保障体系。（发展改革委、财政部、公安部、人力资源社会保障部、卫生健康委、教育部、住房城乡建设部、市场监管总局、医保局、省级有关部门等负责）
　　（五）大力提升农业转移人口就业能力。深入实施新生代农民工职业技能提升计划，加强对新生代农民工等农业转移人口的职业技能培训。支持企业特别是规模以上企业或吸纳农民工较多企业开展岗前培训、新型学徒制培训和岗位技能提升培训，并按规定给予培训补贴。（人力资源社会保障部、教育部、财政部、省级有关部门等负责）
　　（六）加大“人地钱挂钩”配套政策的激励力度。提高城市政府吸纳农业转移人口落户积极性，加大农业转移人口市民化奖励资金支持力度，加大新增建设用地计划指标与吸纳落户数量挂钩力度。维护进城落户农民土地承包权、宅基地使用权、集体收益分配权，不得强行要求其转让上述权益或将此作为落户前置条件；按照依法自愿有偿原则，探索其转让上述权益的具体办法。探索利用大数据技术建立各城市城区常住人口等的常态化统计机制，为政策制定提供支撑。（财政部、自然资源部、农业农村部、发展改革委、统计局、省级有关部门等负责）

　　三、优化城镇化空间格局
　　完善和落实主体功能区战略，发挥各地区比较优势，增强经济发展优势区域承载能力，构建大中小城市和小城镇协调发展的城镇化空间格局，形成高质量发展的动力系统。
　　（七）加快发展重点城市群。加快实施京津冀协同发展、长三角区域一体化发展、粤港澳大湾区建设、长江经济带发展、黄河流域生态保护和高质量发展战略。全面实施城市群发展规划，推动哈长、长江中游、中原、北部湾城市群建设取得阶段性进展，支持关中平原城市群规划实施联席会议制度落地生效，推动兰州-西宁、呼包鄂榆等城市群健全一体化发展工作机制，促进天山北坡、滇中等边疆城市群及山东半岛、黔中等省内城市群发展。（发展改革委、自然资源部、住房城乡建设部、工业和信息化部、交通运输部、生态环境部、省级有关部门等负责）
　　（八）编制成渝地区双城经济圈建设规划纲要。加快推进规划编制实施，促进重庆市、四川省通力协作，加大成渝地区发展统筹力度，发挥中心城市带动作用，加强交通、产业、环保、民生政策对接，共同建设具有全国影响力的科技创新中心，加快培育形成新动力源。（发展改革委、重庆市和四川省有关部门等负责）
　　（九）大力推进都市圈同城化建设。深入实施《[关于培育发展现代化都市圈的指导意见](https://www.pkulaw.com/chl/d247cf138d9f2a4bbdfb.html)》，建立中心城市牵头的协调推进机制，支持南京、西安、福州等都市圈编制实施发展规划。以轨道交通为重点健全都市圈交通基础设施，有序规划建设城际铁路和市域（郊）铁路，推进中心城市轨道交通向周边城镇合理延伸，实施“断头路”畅通工程和“瓶颈路”拓宽工程。支持重点都市圈编制多层次轨道交通规划。（发展改革委、自然资源部、住房城乡建设部、工业和信息化部、交通运输部、生态环境部、国铁集团、省级有关部门等负责）
　　（十）提升中心城市能级和核心竞争力。优化发展直辖市、省会城市、计划单列市、重要节点城市等中心城市，强化用地等要素保障，优化重大生产力布局。完善部分中心城市市辖区规模结构和管辖范围，解决发展空间严重不足问题。（发展改革委、自然资源部、住房城乡建设部、工业和信息化部、民政部、省级有关部门等负责）
　　（十一）推进以县城为重要载体的新型城镇化建设。加快印发指导意见，明确发展目标和建设任务，加大要素保障力度和政策扶持力度，抓紧补上新冠肺炎疫情发生后暴露出来的短板弱项，推进环境卫生设施提级扩能、市政公用设施提挡升级、公共服务设施提标扩面、产业配套设施提质增效。（发展改革委、财政部、住房城乡建设部、省级有关部门等负责）
　　（十二）规范发展特色小镇和特色小城镇。强化底线约束，严格节约集约利用土地、严守生态保护红线、严防地方政府债务风险、严控“房地产化”倾向，进一步深化淘汰整改。强化政策激励，加强用地和财政建设性资金保障，鼓励省级政府通过下达新增建设用地计划指标、设立省级专项资金等方式择优支持，在有条件区域培育一批示范性的精品特色小镇和特色小城镇。强化正面引导，制定特色小镇发展导则，挖掘推广第二轮全国特色小镇典型经验。（发展改革委、自然资源部、财政部、住房城乡建设部、体育总局、林草局、省级有关部门等负责）
　　（十三）推进边境地区新型城镇化建设。在边境地区推进潜力型城镇以产聚人、战略支点型城镇以城聚产，打造以内陆邻近的大中城市为辐射源、边境县级市及地级市市辖区为枢纽、边境口岸和小城镇为节点、边境特色小镇为散点的边境一线城镇廊带。推进兴边富民行动，改善边境一线城镇基础设施和公共服务，建设沿边抵边公路。实施守边固边工程。（发展改革委、财政部、住房城乡建设部、自然资源部、交通运输部、省级有关部门等负责）
　　（十四）推进大型搬迁安置区新型城镇化建设。顺应大型搬迁安置区转向新型城镇化建设新阶段的发展要求，加快推进搬迁人口市民化进程，强化产业就业支撑，帮助搬迁人口尽快解决稳定发展问题，适应新环境、融入新社区。（发展改革委、省级有关部门等负责）
　　（十五）优化行政区划设置。统筹新生城市培育和收缩型城市瘦身强体，按程序推进具备条件的非县级政府驻地特大镇设市，有序推进“县改市”“县改区”“市改区”，稳妥调减收缩型城市市辖区，审慎研究调整收缩型县（市）。全面完成各省（区、市）设镇设街道标准制定工作，合理推进“乡改镇”“乡（镇）改街道”和乡镇撤并。（民政部、发展改革委、省级有关部门等负责）

　　四、提升城市综合承载能力
　　着眼于增强人口经济承载和资源优化配置等核心功能，健全城市可持续发展体制机制，提升城市发展质量。
　　（十六）补齐城市公共卫生短板。改革完善疾病预防控制体系，健全公共卫生重大风险研判、评估、决策、防控协同机制，完善重大疫情预警、救治和应急处置机制，强化重要物资储备，推动城市群、都市圈内城市建立联防联控机制。整治城市环境卫生死角，建立严格检疫、定点屠宰、冷鲜上市的畜禽产品供应体系，健全污水收集处理和生活垃圾分类处理设施。（卫生健康委、发展改革委、应急管理部、工业和信息化部、住房城乡建设部、省级有关部门等负责）
　　（十七）改善城市公用设施。健全城市路网系统，完善公交专用道、非机动车和行人交通系统、行人过街设施。完善市政管网和排水防涝设施。健全停车场、智能快件箱、社区菜市场等便民设施。扩大普惠性养老、幼儿园和托育服务供给。实施全民健康保障工程、全民健身提升工程、智慧广电公共服务工程。（住房城乡建设部、发展改革委、教育部、民政部、卫生健康委、商务部、体育总局、广电总局、省级有关部门等负责）
　　（十八）实施新型智慧城市行动。完善城市数字化管理平台和感知系统，打通社区末端、织密数据网格，整合卫生健康、公共安全、应急管理、交通运输等领域信息系统和数据资源，深化政务服务“一网通办”、城市运行“一网统管”，支撑城市健康高效运行和突发事件快速智能响应。（发展改革委、卫生健康委、公安部、住房城乡建设部、应急管理部、交通运输部、省级有关部门等负责）
　　（十九）加快推进城市更新。改造一批老旧小区，完善基础设施和公共服务配套，引导发展社区便民服务。改造一批老旧厂区，通过活化利用工业遗产和发展工业旅游等方式，将“工业锈带”改造为“生活秀带”、双创空间、新型产业空间和文化旅游场地。改造一批老旧街区，引导商业步行街、文化街、古城古街打造市民消费升级载体，因地制宜发展新型文旅商业消费聚集区。改造一批城中村，探索在政府引导下工商资本与农民集体合作共赢模式。开展城市更新改造试点，提升城市品质和人居环境质量。（住房城乡建设部、发展改革委、民政部、自然资源部、商务部、文化和旅游部、工业和信息化部、农业农村部、省级有关部门等负责）
　　（二十）改革建设用地计划管理方式。推动建设用地资源向中心城市和重点城市群倾斜。鼓励盘活低效存量建设用地，控制人均城市建设用地面积。修改[土地管理法实施条例](https://www.pkulaw.com/chl/eb54177320bcc94bbdfb.html)并完善配套制度，分步实现城乡建设用地指标使用更多由省级政府负责，将由国务院行使的农用地转为建设用地审批权以及永久基本农田、永久基本农田以外的耕地超过35公顷、其他土地超过70公顷的土地征收审批权，授权省级政府或委托试点地区的省级政府实施。探索建立全国性的建设用地、补充耕地指标跨区域交易机制。（发展改革委、自然资源部、省级有关部门等负责）
　　（二十一）改革城市投融资机制。在防范化解地方政府债务风险、合理处置存量债务的前提下，完善与新型城镇化建设相匹配的投融资工具。支持符合条件企业发行企业债券，用于新型城镇化建设项目、城乡融合典型项目、特色小镇和特色小城镇建设项目等。鼓励开发性政策性金融机构按照市场化原则和职能定位，对投资运营上述项目的企业进行综合授信，加大中长期贷款投放规模和力度。（人民银行、银保监会、财政部、发展改革委、开发银行、农业发展银行、省级有关部门等负责）
　　（二十二）改进城市治理方式。推动城市政府向服务型转变、治理方式向精细化转型、配套资源向街道社区下沉。加强和创新社区治理，引导社会组织、社会工作者和志愿者等参与，大幅提高城市社区综合服务设施覆盖率。提高国土空间规划水平，顺应城市发展逻辑和文化传承，落实适用、经济、绿色、美观的新时期建筑方针，加强建筑设计和城市风貌管理，提高城市绿色建筑占新建建筑比重。（民政部、住房城乡建设部、发展改革委、自然资源部、省级有关部门等负责）

　　五、加快推进城乡融合发展
　　突出以城带乡、以工促农，健全城乡融合发展体制机制，促进城乡生产要素双向自由流动和公共资源合理配置。
　　（二十三）加快推进国家城乡融合发展试验区改革探索。指导试验区分别制定实施方案。推动试验区在健全城乡人口迁徙制度、完善农村产权抵押担保权能、搭建城乡产业协同发展平台等方面先行先试，引导县级土地储备公司和融资平台公司参与相关农村产权流转及抵押，加快探索行之有效的改革发展路径。（发展改革委、公安部、自然资源部、人民银行、有关省级部门等负责）
　　（二十四）全面推开农村集体经营性建设用地直接入市。出台农村集体经营性建设用地入市指导意见。允许农民集体妥善处理产权和补偿关系后，依法收回农民自愿退出的闲置宅基地、废弃的集体公益性建设用地使用权，按照国土空间规划确定的经营性用途入市。启动新一轮农村宅基地制度改革试点。（自然资源部、农业农村部、住房城乡建设部、省级有关部门等负责）
　　（二十五）加快引导工商资本入乡发展。开展工商资本入乡发展试点。发挥中央预算内投资和国家城乡融合发展基金作用，支持引导工商资本和金融资本入乡发展。培育一批城乡融合典型项目，形成承载城乡要素跨界配置的有效载体，在长江流域开展生态产品价值实现机制试点。允许符合条件的入乡就业创业人员在原籍地或就业创业地落户并依法享有相关权益。（中央统战部、全国工商联、发展改革委、人民银行、公安部、农业农村部、省级有关部门等负责）
　　（二十六）促进城乡公共设施联动发展。推进实施城乡统筹的污水垃圾收集处理、城乡联结的冷链物流、城乡农贸市场一体化改造、城乡道路客运一体化发展、城乡公共文化设施一体化布局、市政供水供气供热向城郊村延伸、乡村旅游路产业路等城乡联动建设项目，加快发展城乡教育联合体和县域医共体。（发展改革委、住房城乡建设部、教育部、卫生健康委、文化和旅游部、省级有关部门等负责）

　　六、组织实施
　　（二十七）强化部际协同。国家发展改革委依托城镇化工作暨城乡融合发展工作部际联席会议制度，强化统筹协调和指导督促，并总结推广第三批国家新型城镇化综合试点等典型经验。各有关部门要细化制定具体措施，调动本系统力量扎实推进。
　　（二十八）压实地方责任。省级发展改革委要牵头会同省级其他有关部门，结合实际做好组织调度和任务分解。市县级政府要将各项任务落实到事，确保任务落地生效。

© 北大法宝：（[www.pkulaw.com](https://www.pkulaw.com/)) 专业提供法律信息、法学知识和法律软件领域各类解决方案。北大法宝为您提供丰富的参考资料，正式引用法规条文时请与标准文本核对。
欢迎查看所有[产品和服务](https://www.pkulaw.com/helps/2.html)。法宝快讯：[如何快速找到您需要的检索结果？](https://www.pkulaw.com/helps/70.html)[法宝](https://www.pkulaw.com/helps/69.html)V6有何新特色？

扫描二维码阅读原文

**原文链接：**<https://www.pkulaw.com/chl/3724bd876ece5bacbdfb.html>
